# Supplementary material for: Crossing enhanced and high fidelity SpCas9 nucleases to optimize specificity and cleavage
Source: Genome Biol. 2017 Oct 6;18:190. doi: 10.1186/s13059-017-1318-8 (PMC6389135; doi:10.1186/s13059-017-1318-8)
Supplement: Supplementary file 1 — Supplementary results and figures. (DOCX 5322 kb) [file 13059_2017_1318_MOESM1_ESM.docx]

**Table of Contents**

[Supplementary results 1: Compatibility of spacer design practices with the high fidelity nucleases 2](#_Toc488346139)

[Supplementary results 2: Effects of the transfection conditions, nuclease expression levels and post-transfection detection time on the measured activities 4](#_Toc488346140)

[Supplementary Figures 7](#_Toc488346141)

[Figure S1 7](#_Toc488346142)

[Figure S2 9](#_Toc488346143)

[Figure S3 11](#_Toc488346144)

[Figure S4 12](#_Toc488346145)

[Figure S5 14](#_Toc488346146)

[Figure S6 15](#_Toc488346147)

[Figure S7 17](#_Toc488346148)

[Figure S8 19](#_Toc488346149)

[Figure S9 20](#_Toc488346150)

[Figure S10 21](#_Toc488346151)

[Figure S11 23](#_Toc488346152)

# **Supplementary results 1: Compatibility of spacer design practices with the high fidelity nucleases**

We systematically examined the compatibility of these SpCas9 variants with the routinely used practices employed in sgRNA spacer design to comply with the preferences for a G nucleotide as transcription initiation site for the human U6 promoter for those targets that would otherwise require a non-G as the 5’-end nt of a 20 nt-long spacer.

**Appending an extra 5' G nucleotide**

To assess systematically the effects of an appended extra 5' G to the 20-nt spacer on the performances of the eSpCas9 [1] and SpCas9-HF1 [2] variants, first, we selected 26 target sequences for EGFP and targeted each of them in parallel with two sgRNAs bearing either a 20 nt-long spacer starting with a 5' G matching nucleotide or with the same spacer but extended with a *mismatching* extra 5' G (that is a 21 nt-long spacers with 5' GG dinucleotide), and tested the nucleases in EGFP disruption assay in N2a.EGFP cells. We found that adding a mismatching starting G in 21 nt-long spacers, while causing no dramatic effect on the performance of the WT nuclease, diminishes the activities of both mutant nuclease variants in a target dependent manner (Fig. 2a and Additional File 1: Figure S2a, c): only on 7 (eSpCas9) and 4 (SpCas-HF1) targets out of 26 they are able to attain 80% and 50% activity, respectively, of that without the extra G (Additional File 1: Figure S2a, c). Interestingly, the same four targets were cleaved least by both nucleases with 21 nt-long spacers. The extra G completely abolishes the activity of HeFSpCas9 on all of the four targets that it cleaves with 20 nt-long spacers (data not shown).

Next, we examined whether the nuclease variants would tolerate better the extra 5' G in their sgRNAs when that is matching to the corresponding target nucleotide. We performed experiments with another set of 20 sgRNAs, where half of them possessed a matching starting G (20-nt spacers) and the other half are the same spacers with a *matching* extra G appended at their 5'-end (21 nt-long spacers). While it does not affect the WT SpCas9, we found that the *matching* extra G diminishes even more the activities of eSpCas9 and SpCas9-HF1, leaving only 4 and 2 targets cleaved to a detectable extent, respectively (Fig. 2b and Additional File 1: Figure S2b, d). Altogether, these results indicate that appending an extra 5' G to the spacer sequence, is not routinely applicable with these variants of SpCas9s.

**Applying 20 nt-long spacers with mismatching 5' G or with a non-G nucleotide**

The other common practice used for targets that would require sgRNAs with a starting non-G nucleotide is to either change the first nucleotide of the sgRNA to a mismatching G or to just use it without alteration, i.e. with a starting non-G nucleotide. In the latter case the RNA polymerase starts to transcribe efficiently the sgRNA when encounters the first suitable nucleotide (thought to be primarily a G). Thus, the majority of the sgRNAs in this case will be shorter than 20 nt-long, with lengths depending on their 5'-end sequences. Using 18 sgRNAs, half of them containing a 5' matching non-G nucleotide and the other half are the same spacers but with a G mutation at this position (thus, these latter spacers are 20 nt-long and their first nucleotides are a mismatching G), revealed that the WT SpCas9 works slightly better with a mismatching starting G as compared to with shorter spacers (Additional File 1: Figure S2e). However, while eSpCas9 works relatively well with these shorter guides, showing more than 80% activity of that of WT SpCas9 on the majority of the targets (6/9), the mismatching G considerably decreases its activity (Additional File 1: Figure S2e). SpCas9-HF1 shows substantially decreased activity with both sets of sgRNAs in accord with expectations [2].

**Using 17, 18 and 19 nt-long truncated spacers**

To see how SpCas9 variants can tolerate shorter guides, 5 targets were chosen (they are also on Fig. 3a) to generate 9 truncated 17, 18 or 19 nucleotide-long spacer-containing guides, all starting with a matching G. Eight out of the nine truncated spacers resulted cleavage of the targets by the WT (Additional File 1: Figure S2f). By contrast, eSpCas9 and SpCas9-HF1 cleaved only three and one targets, respectively, with all being 19 nt-long spacers. HeFSpCas9 cleaved no truncated targets. These results are in line with earlier observations [1, 2]. Thus, these experiments employing 100 sgRNAs on 49 target sites show that high fidelity/enhanced nucleases can be routinely used only with perfectly matching 20 nt-long spacers.

# **Supplementary results 2: Effects of the transfection conditions, nuclease expression levels and post-transfection detection time on the measured activities**

We examined the effect of various expression conditions on the experimental outcomes in these studies. The mutations in increased fidelity SpCas9 nucleases (eSpCas9 and SpCas9-HF1) are designed to decrease off-target effects. Since reducing the amount of the nuclease is an alternative strategy for this purpose, we considered it to be very important to distinguish which of the two strategies applies better to eSpCas9 and SpCas9-HF1 and carried out the following experiments.

In the original studies, the mutations created did not seem to interfere with the stability or steady state expression level of either eSpCas9 [1] or SpCas9-HF1 [2], however, in these studies the two nucleases were expressed in different cells and were compared to the WT SpCas9s employing different WT protein-expressing vectors. Here, by analyzing side by side the wild type and the two mutant proteins, SpCas9-HF1 reached considerably lower expression levels than WT and eSpCas9 did (data not shown). In order to facilitate a more accurate comparison, we have cloned SpCas9-HF1 to the same plasmid backbone and have tailored to possess the same NLS and FLAG tag at its termini as the WT and the eSpCas9 nucleases. Although the expression level of SpCas9-HF1 has increased by this, as compared to its expression from the original plasmid (Additional File 1: Figure S5a) it still does not reach the expression level of eSpCas9 (Additional File 1: Figure S5b). To verify if the lower level of expression is associated to some differences outside the coding region of the protein, we subcloned its ApaI-SacI fragment of SpCas9-HF1 that contains all concerned mutation, to the corresponding segment of HeFSpCas9 expressing vector. However, we find that the subcloning does not result in an increased expression level (Additional File 1: Figure S5c) ruling out that the presence of an unnoticed mutation in the vector backbone would be responsible for its lower level of expression.

The differing expression levels of eSpCas9 and SpCas9-HF1 pose the question whether which of the following approaches cause less obscurities to their comparison: the altered expression levels measured at day 3 post-transfection when using identical amount of plasmid DNA for transfection, or the differing decay characteristics of the transfected DNA when employing differing amounts of plasmid DNA in order to reach more similar expression levels for the nucleases, at day 3.

Using differing amounts of nuclease-expressing plasmid for transfection (i.e., 1, 1/3 or 1/6 unit) while keeping the total transfected DNA amounts identical by using a mock plasmid of identical size, we found no difference in the respective EGFP-disruption percentages for either WT or eSpCas9 (Additional File 1: Figure S6a). These varying amounts of nuclease-expression-plasmid DNAs for WT and eSpCas9 resulted protein expression levels that encompassed those reached by SpCas9-HF1 when using its higher (i.e., 1 unit) plasmid DNA amount (Additional File 1: Figure S5d). Since the expression of SpCas9-HF1 falls between the expression levels reached by transfecting identical or one third fractional amount of plasmid DNA for eSpCas9 or the wild type protein (Additional File 1: Figure S5d), we compared the activities of WT, eSpCas9 and SpCas9-HF1 on ten targets using either equally higher, or 1/3 fractional amounts of these nuclease-expression-plasmids for transfection. The results showed that, the primary factors determining the relative (to wild type) efficiencies of the nuclease variants are their intrinsic activities toward these targets rather than the expression levels achieved from these expression-vectors or the differing decay characteristics of the transfected plasmid DNA, which modulate only moderately the activities of these nucleases at the applied conditions (Additional File 1: Figure S6b and c).

We have also examined the effects of lower expression levels on the off-target activities of these nucleases programmed with partially mismatching sgRNAs on ten positions using 5 targets (also investigated on Figure 4a) using EGFP disruption assay. Additional File 1: Figure S6g shows that for each nuclease the off-target:on-target ratio varies from little to none with differing amounts of plasmid DNAs used.

These results further confirm that employing these nuclease-expressing vector constructs and transfecting the cells with either identical nuclease-expression-plasmid amounts (while reaching differing expressions) or with differing amounts (while reaching more similar expressions), cause less modulation in the observed relative activities and specificities of the high fidelity and enhanced nucleases, as compared to those differences that are caused by their intrinsic characteristics.

The EGFP disruption experiments are conducted in this study on a 48-well-plate format. We found that the transfection efficiencies varied little among the wells of one plate, but did vary more among plates, especially, when transfections were made at different days. We decided to examine also the effects of the varying transfection efficiencies on the activities of the nucleases in EGFP disruption assay. We compared the results of the experiments found when high (between 90 and 98%) and when low (between 50 and 75%) transfection efficiencies were achieved involving 10 identical targets. The actual disruption values observed seem to depend very much on the transfection efficiency, however, the relative activities (compared to the wild type SpCas9) are not significantly affected, as indicated by the overlapping confidence intervals for the medians and the comparison of means, on either day 3 or 7 post-transfection, within these transfection efficiency ranges examined (Additional File 1: Figure S6d, e and Additional File 4: Statistics). Thus, the differing intrinsic activities of eSpCas9 and SpCas9-HF1 nucleases relative to the WT protein are apparent independently of the transfection efficiencies within the range achieved here. Nevertheless, these results highlight the importance to carry out the particular experiments side by side, on the nuclease variants and WT to be compared reaching similar transfection efficiencies.

We measured the disruption efficiencies at 3^rd^ and 7^th^ days post-transfection. Additional File 1: Figure S6f compiles the data from four experiments conducted at various conditions with SpCas9 and its high fidelity and enhanced variants. Somehow larger differences among the nucleases can be detected on the 3^rd^ rather than on the 7^th^ days. This is likely due to the nucleases (WT and sometimes the eSpCas9) reaching close to saturation levels of EGFP disruption earlier than the 7^th^ day. Thus, the 3^rd^ day post-transfection data are more sensitive reporters of the intrinsic activities of these nucleases and therefore, we used the 3^rd^ day data to evaluate their on-target activities. By contrast, the 7^th^ day post-transfection data are more likely to include the full spectrum of the off-target activities and therefore, we used the 7^th^ day post-transfection data to compare the off-target activities of the nucleases.

In most of the other assays used in the literature and in our laboratory, the effect of transiently transfected SpCas9 is usually minimal after the third day post-transfection. The seemingly longer activity-window detected here is likely attributable to the high stability/slow turnover of EGFP.

# **Supplementary Figures**

## **Figure S1**


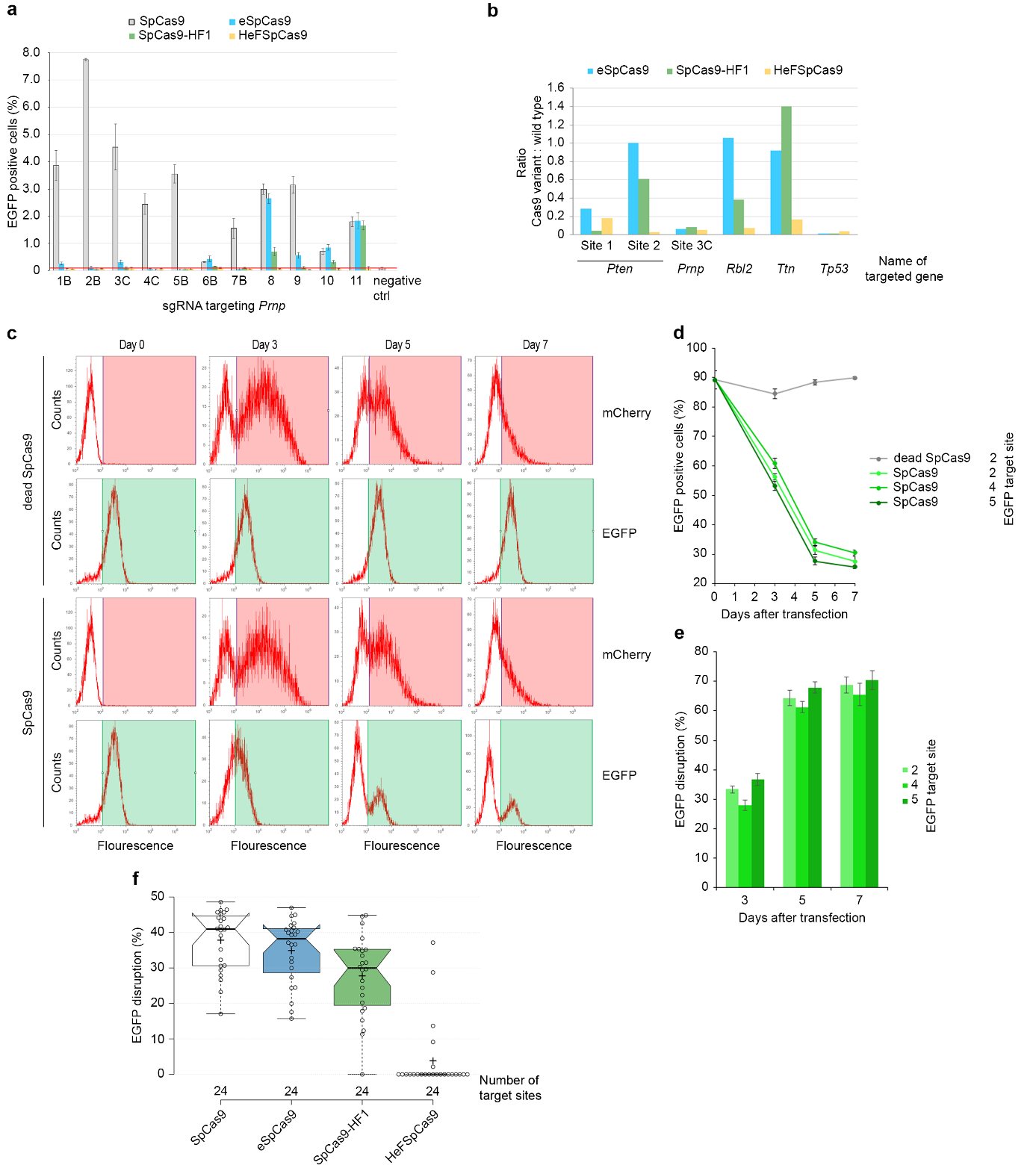


**Figure S1. Indel formation, homology driven integration of donor DNA and EGFP disruption induced by SpCas9 nuclease and its variants. a**, Integration of a donor EGFP cassette without a promoter and equipped with homology arms to the flanking sequences of the coding region of the *Prnp* gene mediated by the SpCas9 nuclease variants. **b**, Indel forming activities of SpCas9 variants compared to the wild type nuclease assessed by TIDE. **c**, EGFP and mCherry FACS histograms of N2a.EGFP reporter cells co-transfected with mCherry/sgRNA expressing plasmid and either dead (targeted to EGFP site 2) or active SpCas9s (targeted to EGFP site 2) expressing plasmids, analyzed on days 0, 3, 5 and 7 post-transfection by flow cytometry. **d**, Percentages of *EGFP positive cells* at various days post-transfection derived from FACS analysis of samples transfected by either dead SpCas9 targeting site 2, or active SpCas9s targeting EGFP sites: 2, 4 or 5. **e**, *EGFP disruption activities* of wild type SpCas9s (targeted to EGFP sites 2, 4 or 5) measured on Day 3, 5 and 7 post-transfection and calculated as described in Methods. **f**, Summary of on-target disruption activities of SpCas9 nucleases. Tukey-type notched boxplots by BoxPlotR: center lines show the medians; box limits indicate the 25^th^ and 75^th^ percentiles; whiskers extend 1.5 times the interquartile range from the 25^th^ and 75^th^ percentiles; notches indicate the 95% confidence intervals for medians; crosses represent sample means; data points are plotted as open circles. The sample points for each nuclease correspond to the data on Figure 3a obtained on 24 targets (Sites targeted are provided in Additional File 2). Statistically different pairs of means at the p<0.05 level: SpCas9 – SpCas9_HF1 (.003); SpCas9 – HeFSpCas9 (<.001); SpCas9_HF1 – HeFSpCas9 (<.001); HeFSpCas9 – eSpCas9 (<.001).

## **Figure S2**

##
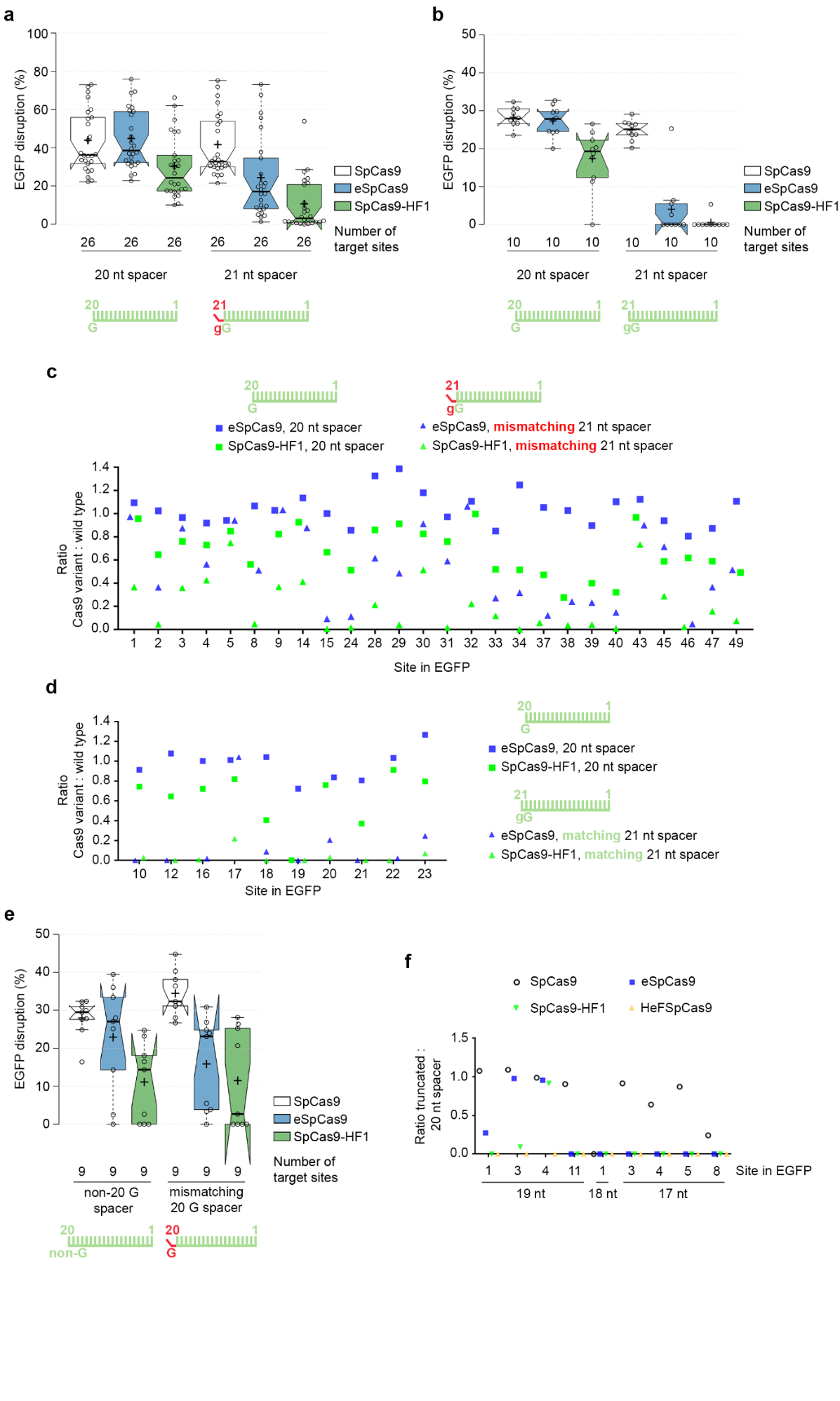


**Figure S2. Effects of various modifications of the sgRNA spacer sequence on the disruption activities of SpCas9 nuclease variants.** Effect of 5' extension of the spacer with **a, c,** a mismatching G nucleotide and **b, d,** a matching G nucleotide on the nuclease activities as compared to those of perfectly matching 20 nt-long spacers. **e**, Effects of 20 nt-long 5' matching non-G and 20 nt-long 5' mismatching G spacers on the nuclease activities. Tukey-type notched boxplots by BoxPlotR: center lines show the medians; box limits indicate the 25^th^ and 75^th^ percentiles; whiskers extend 1.5 times the interquartile range from the 25^th^ and 75^th^ percentiles; notches indicate the 95% confidence intervals for medians; crosses represent sample means; data points are plotted as open circles. The sample points for each nuclease correspond to the number of targets used. Spacers used for the corresponding symbols are depicted as green color combs and the 21^st^ G nucleotide extensions (numbered from PAM sequence) are shown as a red color bent end teeth if mismatching; lower case g-s represent appended nucleotides. **f**, Effect of 5' truncation of sgRNAs on the activities of the wild type SpCas9, eSpCas9, SpCas9-HF1 and HeFSpCas9. Statistically different pairs of means at the p<0.05 level for panels a, b and e that are relevant, are as follows: a, eSpCas9_21mmG – eSpCas9_20G (<.001), SpCas9-HF1_21mmG – SpCas9-HF1_20G (<.001) b, SpCas9-HF1_21G – SpCas9-HF1_20G (<.005). Of note, wild type SpCas9 is not significantly affected by either of the spacer-modifications tested (panels a-e). (Details of statistics on data are provided in Additional File 4.)

**Figure S3**


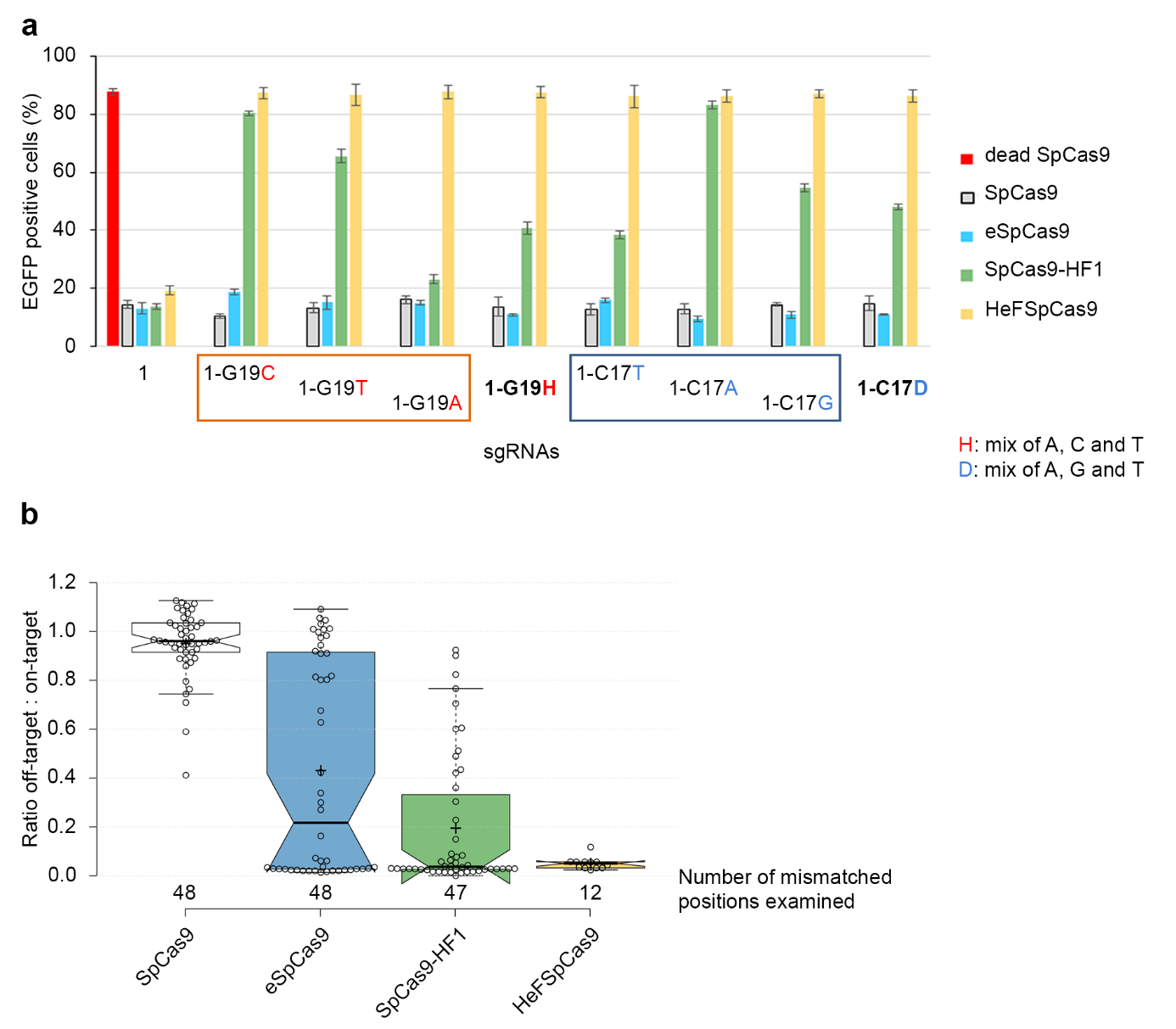


**Figure S3. Disruption and indel formation activities** **of SpCas9 nucleases when programmed with perfectly matched or partially mismatched sgRNAs.**

**a,** EGFP disruption experiments with sgRNAs mismatched at identical positions loaded to SpCas9 nucleases individually or as a mixture of the three possible mismatching variants. **b**, Summary of off-target disruption activities of SpCas9 nucleases. Tukey-type notched boxplots by BoxPlotR: center lines show the medians; box limits indicate the 25^th^ and 75^th^ percentiles; whiskers extend 1.5 times the interquartile range from the 25^th^ and 75^th^ percentiles; notches represent 95% confidence intervals for medians; crosses represent sample means; data points are plotted as open circles; the number of data points, 48 (or 47 for SpCas9-HF1 and 12 for HeFSpCas9) correspond to the number of total mismatched positions examined. (Data used for analysis are from experiments presented on Figure 4a). Statistically different pairs of means at the p<0.05 level: SpCas9 – eSpCas9 (<.001), SpCas9 – SpCas9-HF1 (<.001), SpCas9 – HeFSpCas9 (<.001), SpCas9-HF1 – eSpCas9 (.012), SpCas9-HF1 – HeFSpCas9 (.009), HeFSpCas9 – eSpCas9 (<.001).

## **Figure S4**


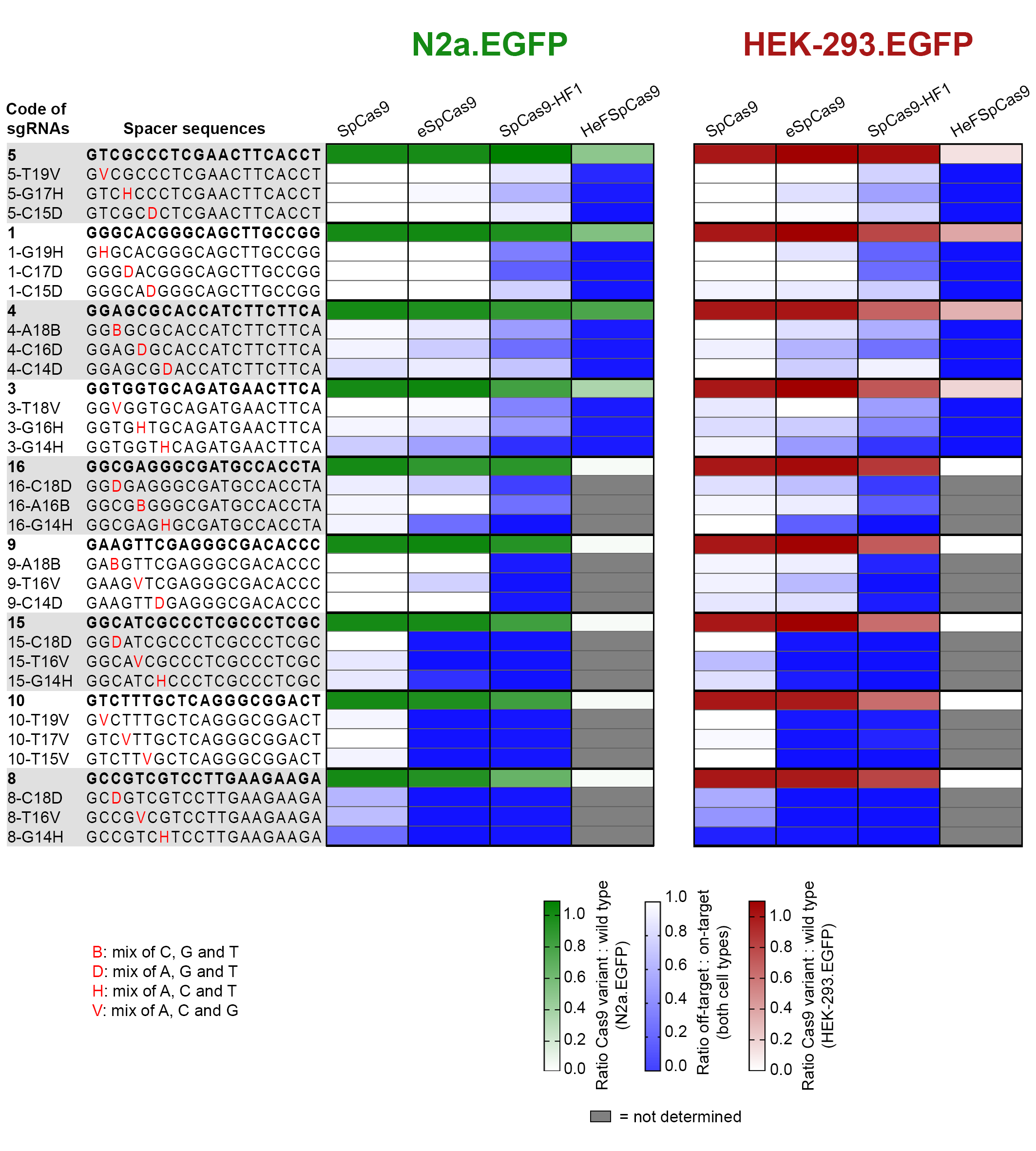


**Figure S4. The same cleavability-ranking of the targets by the nuclease variants and the same fidelity ranking (eSpCas9<SpCas9-HF1<HeFSpCas9) of the nucleases are apparent in HEK-293.EGFP and N2a.EGFP cells.**

Disruption and indel formation activities of SpCas9 nucleases programmed with perfectly matching or partially mismatching sgRNAs. Heat maps are showing the relative activities (white to green in N2a.EGFP cell line and white to red in HEK-293.EGFP cell line) of the nuclease variants compared to the wild type for each of the targets and the ratios of off-target to on-target disruption activities of the wild type and mutant nucleases (blue to white for both cell lines), measured employing the indicated target and mismatching spacer sequences in the two cells; grey boxes: not determined due to diminished on-target activities. Both parameters, disruption ratios to the wild type and the on-target/off-target values, show similar patterns for a given target across the order of nuclease variants in the two types of cells.

## **Figure S5**


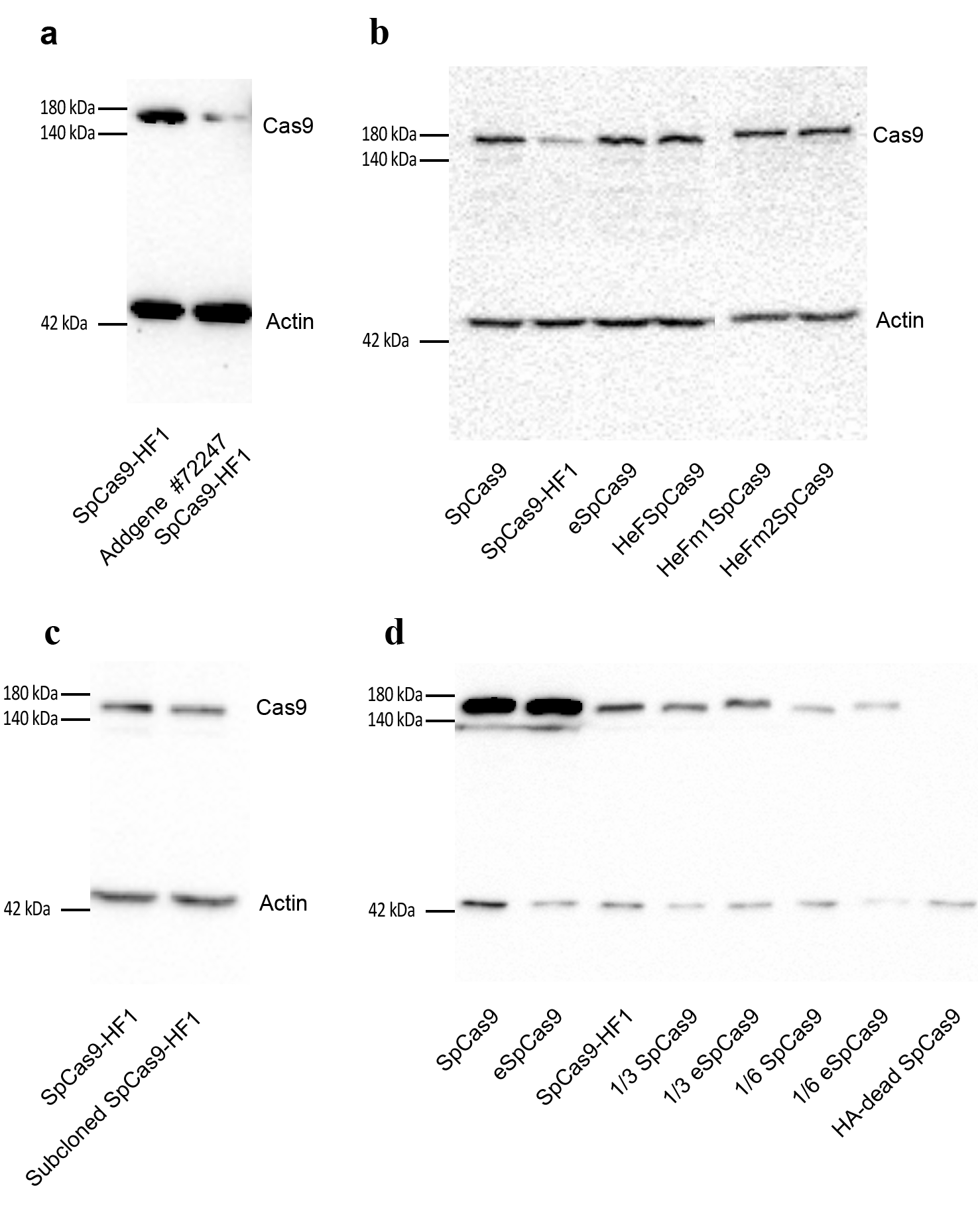


**Figure S5.** **Assessing the expression levels of the nucleases.**

**a**, **b,** **c** and **d**, Immunoblot analyses of the SpCas9 nuclease expression levels in cell lysates of reporter N2a.EGFP cells transfected with the indicated nuclease constructs. The lysates originate from 8-8 independent transfections and were mixed before analysis; cells were harvested at the 3^rd^ day post-transfection. Cells were transfected by equal amounts of total plasmid DNA and also by varying nuclease-expression plasmid amounts: 1, 1/3 and 1/6 fractional amounts in case of the indicated samples. For SpCas9-HF1, giving somewhat lower amounts of expression as compared to the wild type or eSpCas9, the expressions of **a,** the original plasmid (VP12, Addgene # 72247) and **c,** of a subcloned version are also tested. More details are described in the Supplementary results 2.

## **Figure S6**


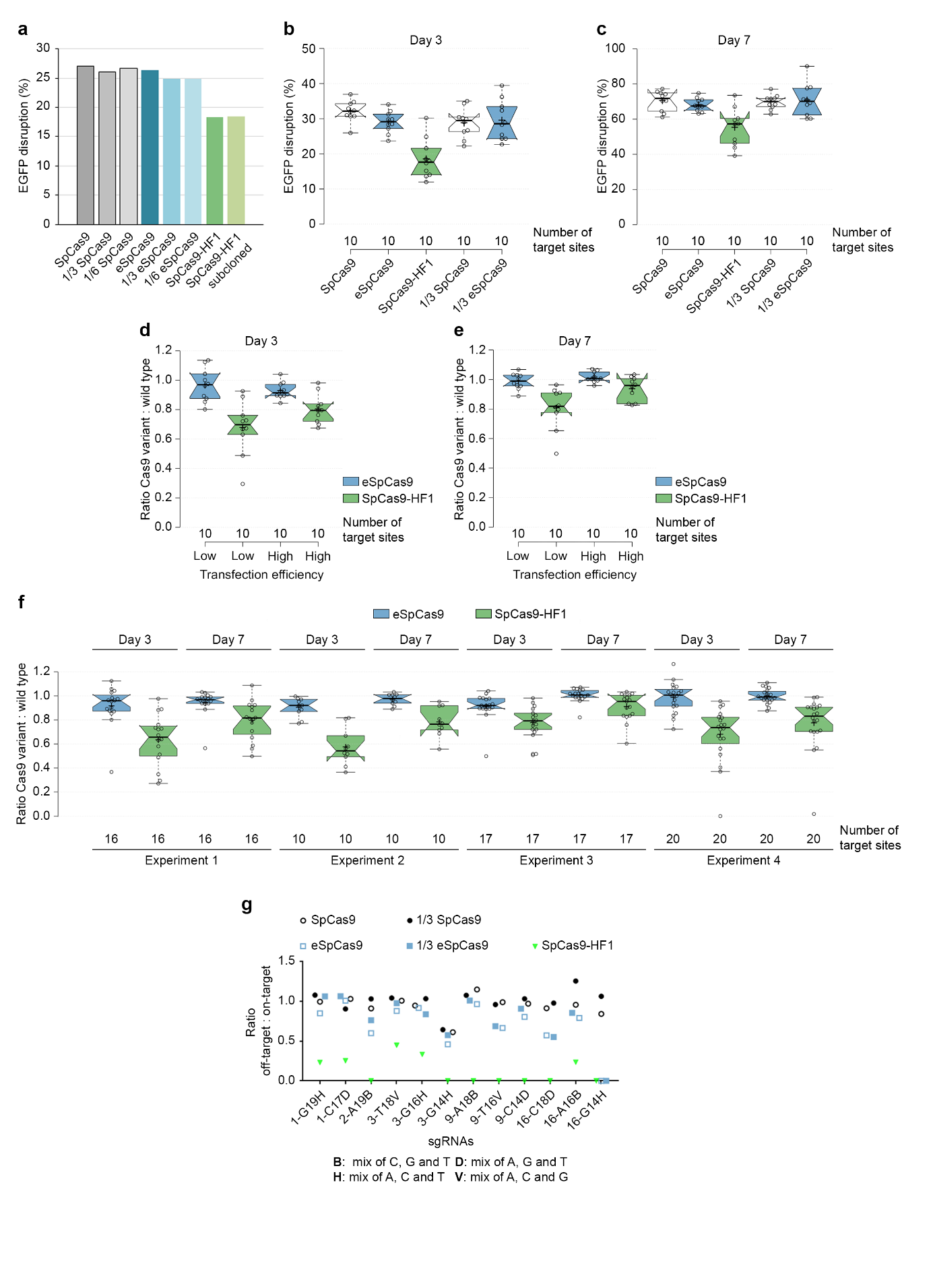


**Figure S6. Effects of the amount of transfected plasmid DNA, transfection efficiencies and post-transfection detection time on the expression levels and/or activities of SpCas9 variants.**

The lower on-target activity of SpCas9-HF1 is more of an intrinsic characteristic of the nuclease rather than being the effect of varying expression levels in the range used. **a,** On-target EGFP disruption activities of wild type, eSpCas9 and SpCas9-HF1 and of 1, 1/3 or 1/6 fractional amounts of wild type and eSpCas9 nucleases at day 3 after transfection of N2a.EGFP cells. The bars represent cells mixed from 8-8 wells before analysis EGFP target site 3 was targeted. **b, c,** On-target EGFP disruption activities of wild type, eSpCas9 and SpCas9-HF1 and of 1 and 1/3 fractional amounts of wild type and eSpCas9 nucleases at day 3 (**b**) and day 7 (**c**) post-transfection of N2a.EGFP cells. **d, e,** Disruption activities of variants eSpCas9 and SpCas9-HF1 compared to the wild type nuclease SpCas9 at day 3 (**d**) and day 7 (**e**) post-transfection when different transfection efficiencies (low and high) are used in N2a reporter cells. **f**, Comparisons of ratios of on-target disruption activities of mutants to wild type nuclease at day 3 and day 7 compiled from various experiments. Tukey-type notched boxplots by BoxPlotR: center lines show the medians; box limits indicate the 25^th^ and 75^th^ percentiles; whiskers extend 1.5 times the interquartile range from the 25^th^ and 75^th^ percentiles; notches represent 95% confidence intervals for medians; crosses represent sample means; data points are plotted as open circles. Sample points correspond to data averages of three parallels for each of the targets. **g**, Ratios of off-target to on-target disruption activities of wild type, eSpCas9 and SpCas9-HF1 and of 1/3 amounts of wild type and eSpCas9 nucleases programmed with partially mismatching sgRNAs as measured at day 7. These single-base mismatching sgRNAs are also tested in Figure 4a.

## **Figure S7**


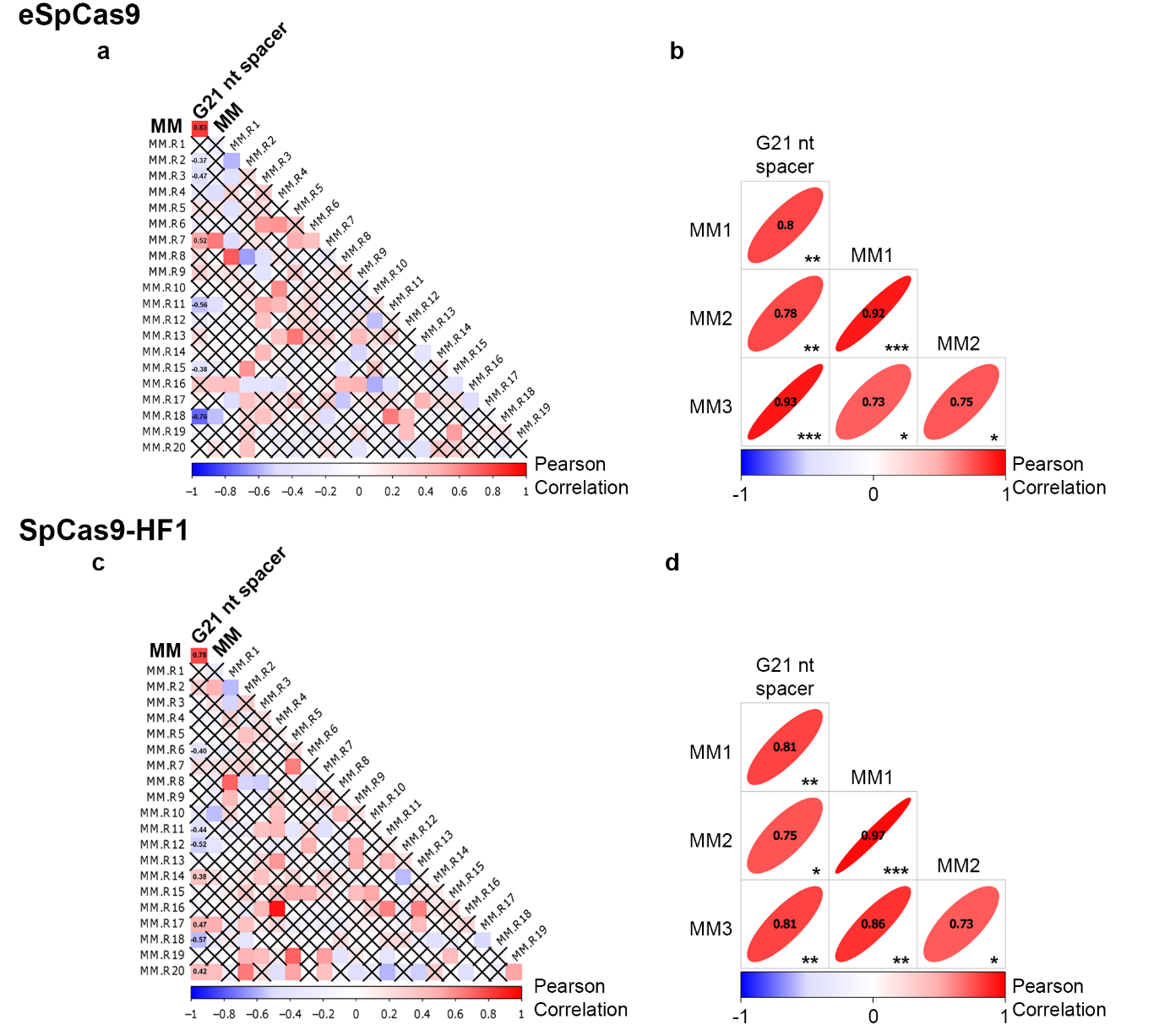


**Figure S7. Correlation between the effects of 5' extension with a mismatching G (21 nt-long) and of single-base mismatches of the sgRNA spacers on the activities of the nuclease variants eSpCas9 and SpCas9-HF1.** Disruption activity values normalized to wild type of (**a,** **c**) eSpCas9 and of (**b,** **d**) SpCas9-HF1 were analyzed when the nucleases are loaded with sgRNAs containing spacers with either a 5' mismatching G-extension, i.e 21 nt-long spacer (G21 nt spacer) or with 20 nt-long spacers bearing one mismatching nucleotide (“MM”) in the sequence where the sgRNAs of the three possible mismatches for a position are pooled, and mismatches at three positions are examined, together (in average, a, c: MM) or separately (b, d: MM1, 2 or 3) . Values are obtained on 10 targets each with nine one-mismatch containing 20 nt- and one 21 nt-long 5' mismatching G spacer (total of 100 spacers). Correlation matrix analysis with significance was done by using R software and the package "corrplot". Numbers represent Pearson correlation coefficients; ellipses on c, d, are the 95% confidence intervals of the correlation coefficients. 2-tailed test of significance is used. ***: p ≤ 0.001, **: p ≤ 0.01, *: p ≤ 0.05. **a,** **c,** Correlation tested between values corresponding to the same target ("G21 nt spacer" vs. "MM ") and when the mismatch-activity values are randomly shuffled (MM.R1-MM.R20) between targets. Insignificant values with p>0.05 are marked by X at the corresponding positions; respective correlation values are added to the first columns. **b, d,** Correlation analysis on the activity values obtained within the three mismatching positions (“MM1-3”) and the 5 G'-extension of the same target (G21 nt spacer), for the 10 targets.

## **Figure S8**


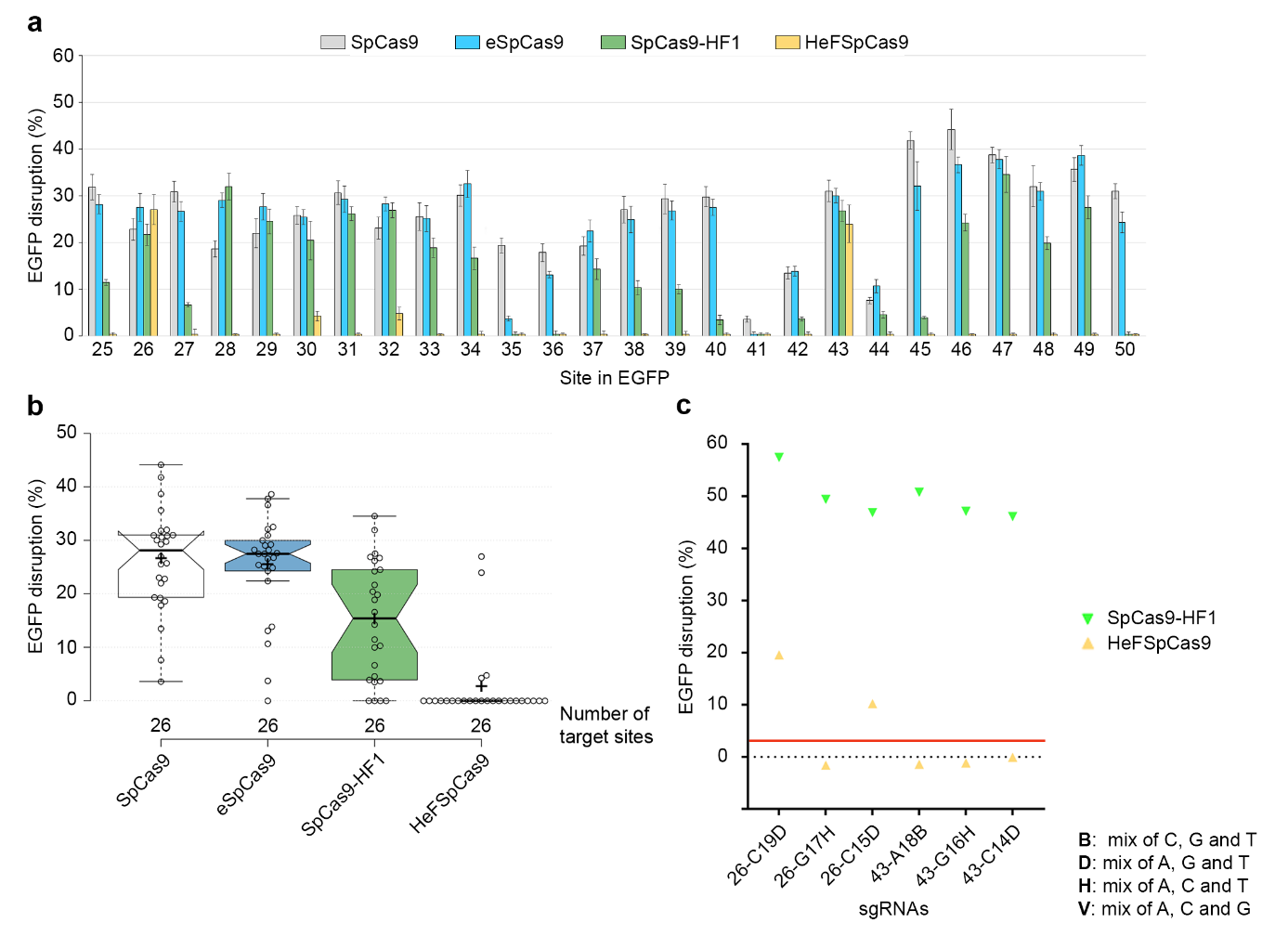


**Figure S8. Disruption activities of SpCas9 nuclease variants when programmed with perfectly matched or partially mismatched sgRNAs.**

**a,** EGFP disruption activities of the nucleases measured for an extended set (compared to Fig. 3a) of additional 26 targets. Values were calculated as described in Methods. Bars correspond to averages of n=3 parallel samples; error bars represent the standard errors estimated by Gaussian error propagation of the component standard deviations (s.d.-s) associated to both EGFP and mCherry (transfection control) values. **b,** Boxplots summarizing the distribution properties of on-target disruption activity data obtained on the additional set of 26 targets from S8a. Tukey-type notched boxplots by BoxPlotR: center lines show the medians; box limits indicate the 25^th^ and 75^th^ percentiles; whiskers extend 1.5 times the interquartile range from the 25^th^ and 75^th^ percentiles; notches represent 95% confidence intervals for medians; crosses represent sample means; data points are plotted as open circles. Statistically different pairs of means at the p<0.05 level: SpCas9 – SpCas9-HF1 (.004), SpCas9 – HeFSpCas9 (<.001), SpCas9-HF1 – eSpCas9 (.009), HeFSpCas9 – eSpCas9 (<.001), SpCas9-HF1 – HeFSpCas9 (<.001). The sites targeted are provided in Additional File 2. **c,** Off-target activity comparisons for SpCas9-HF1 and HeFSpCas9 at day 7. Red line represents background level (3%).

## **Figure S9**


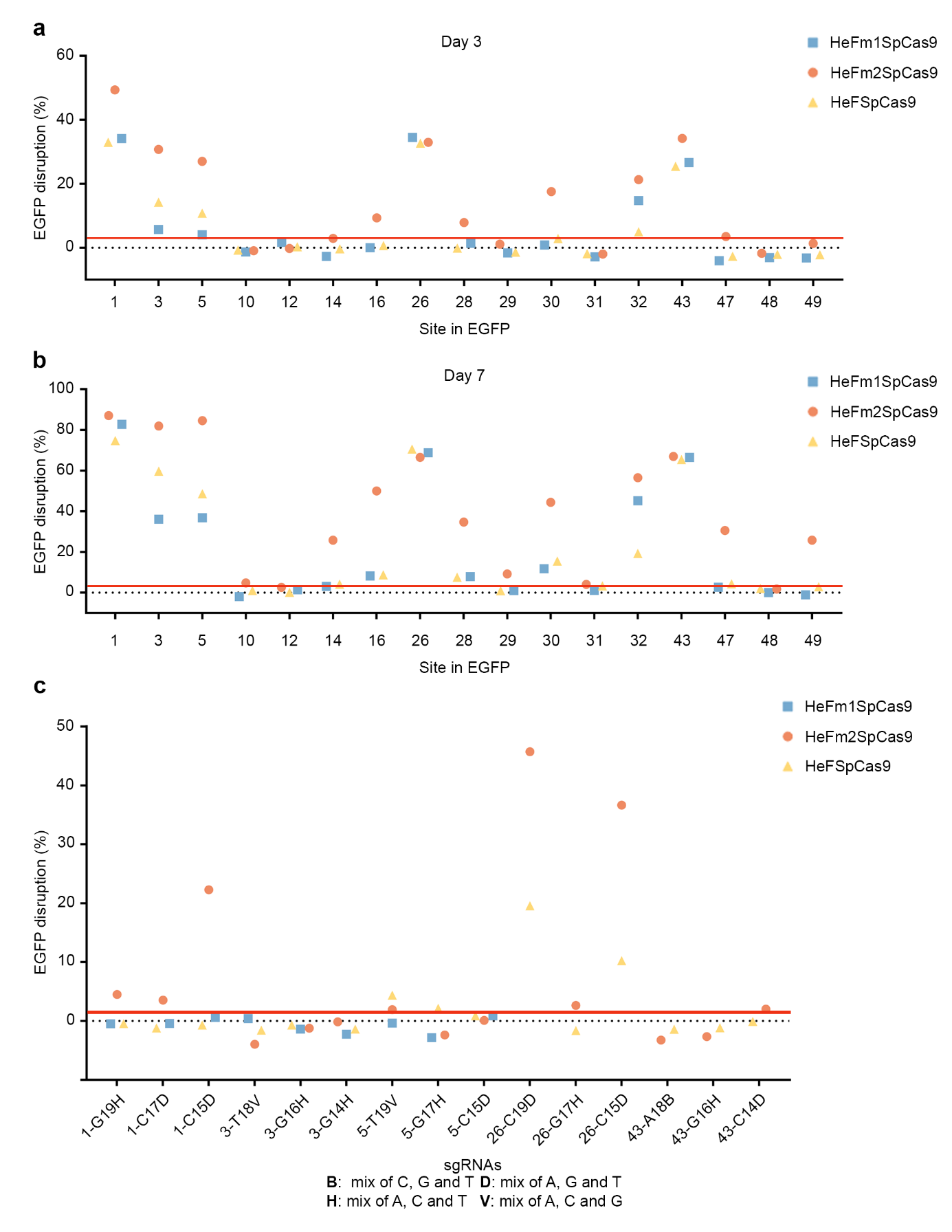


**Figure S9. Disruption activities of HeFSpCas9 nuclease variants when programmed with perfectly matched or partially mismatched sgRNAs. a,** and **b,** on-target disruption activity at day 3 and day 7 post-transfection, respectively; **c**, off-target activity comparisons at day 7. Red line represents background level (3%).

## **Figure S10**


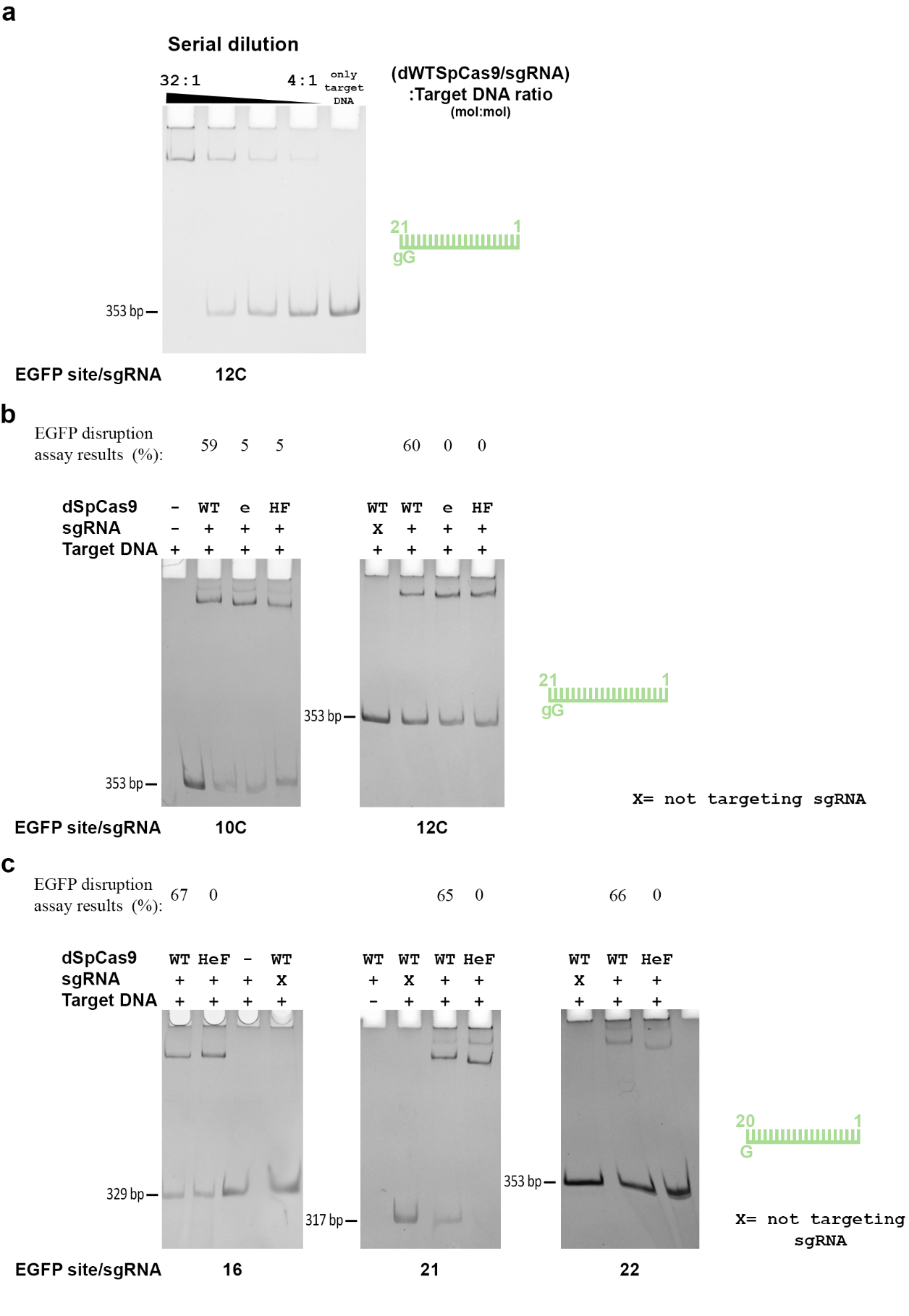


**Figure S10. In vitro DNA binding of the SpCas9 nuclease variants to inefficiently-cleaved targets.**

**a,** Serial dilution of the dead WT SpCas9-sgRNA complex between the range of 32 to 4-fold of the molar amount of PCR amplified DNA target (for details see Methods). **b,** In vitro binding of eSpCas9 and SpCas9-HF1 charged with 21 nucleotide-long sgRNAs to selected targets that were only cleaved by the nuclease variants when the same but only 20 nucleotide-long spacers were applied in the disruption assay. The SpCas9-sgRNA complex:target DNA molar ratio is 4:1 that is a sensitive condition to report on the binding activities (in contrast to the 32:1 ratio where the binding is under saturated condition). **c,** In vitro binding of WT and HeFSpCas9 charged with 20 nucleotide-long sgRNAs that are cleaved efficiently by WT SpCas9 but not by HeFSpCas9 (the SpCas9-sgRNA complex:target DNA molar ratio is the same, 4:1). X indicates sgRNAs that do not target the DNA.

## **Figure S11**


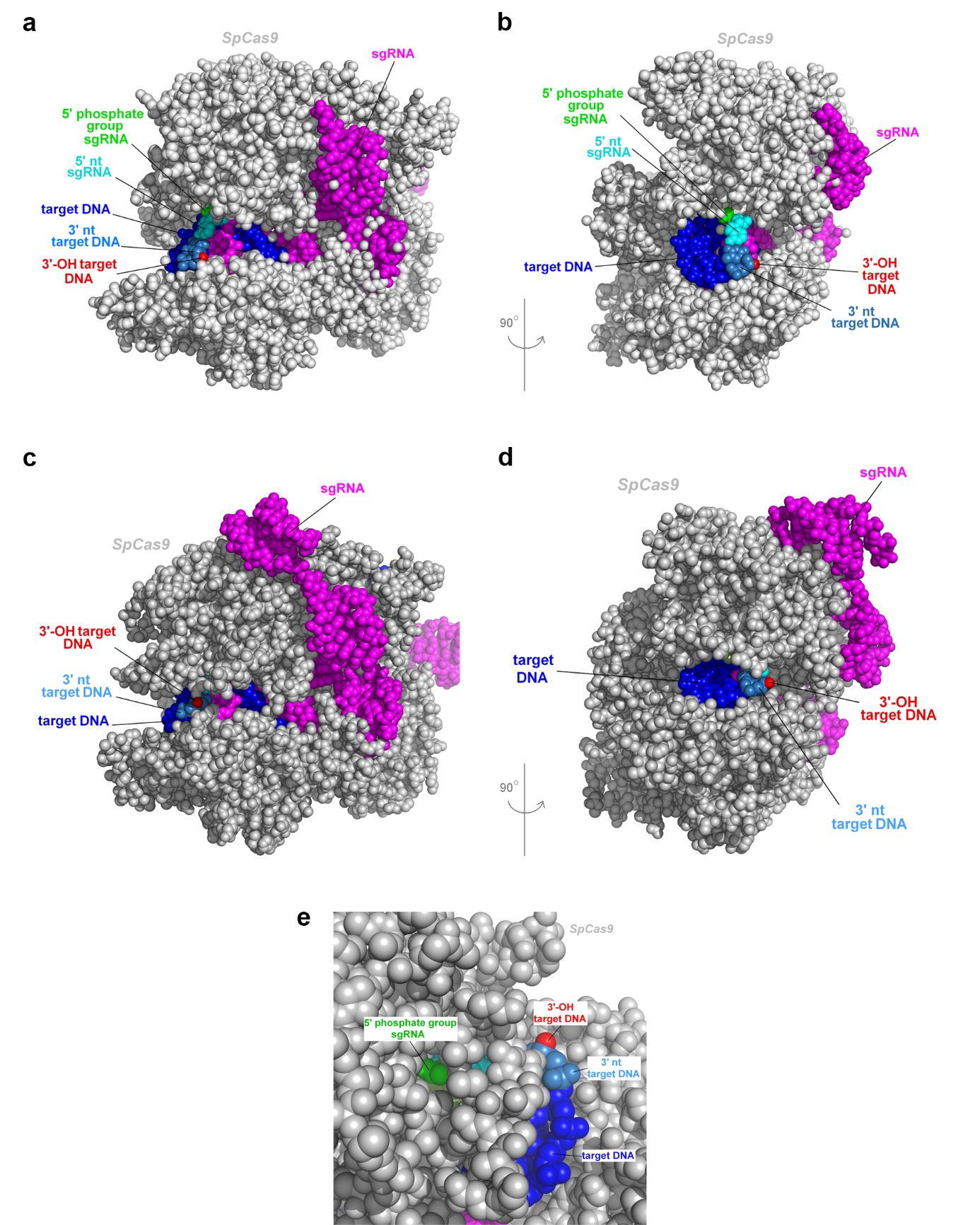


**Figure S11.** **Structural rearrangements taking place in the cleavage-competent state bury the 5’-end of the sgRNA more within the protein’s structure as compared to when in a binding-competent state.**

X-ray structures of SpCas9 complexed with an sgRNA and dsDNA oligos. **a, b**, binding-competent complex (PDB num.: 4un3 [3]). **c, d,** rearranged structure for cleavage (PDB num.: 5f9r [4]); the non-targeted strand is longer with 9 nt and the sgRNA contains the SL3 and an extended RAR region in the latter structures. Structures are presented as sphere-models with colors as follows: protein and non-targeted DNA grey; targeted DNA strand blue with the last 3'-nucleotide in sky-blue and the 3'-OH end in red; sgRNA is magenta with the 5'-end nucleotide in cyan and the 5' phosphate group in green. The sgRNA (magenta) including its 5’ nt (cyan) is more buried in the rearranged structure for cleavage (c, d). The ends of the sgRNA (5’ phosphate group, green) and the target DNA strand (3’-OH group, red) in the cleavage-competent state are taken apart by protein segments (**e**) suggesting that the two strands may exit the protein at separated positions (inferring also that a matching G extension may diminish more the activity than a mismatching one, causing larger distortions to the structure).

1. Slaymaker IM, Gao L, Zetsche B, Scott DA, Yan WX, Zhang F: **Rationally engineered Cas9 nucleases with improved specificity.** *Science* 2016, **351:**84-88.

2. Kleinstiver BP, Pattanayak V, Prew MS, Tsai SQ, Nguyen NT, Zheng Z, Joung JK: **High-fidelity CRISPR-Cas9 nucleases with no detectable genome-wide off-target effects.** *Nature* 2016, **529:**490-495.

3. Anders C, Niewoehner O, Duerst A, Jinek M: **Structural basis of PAM-dependent target DNA recognition by the Cas9 endonuclease.** *Nature* 2014, **513:**569-573.

4. Jiang FG, Taylor DW, Chen JS, Kornfeld JE, Zhou KH, Thompson AJ, Nogales E, Doudna JA: **Structures of a CRISPR-Cas9 R-loop complex primed for DNA cleavage.** *Science* 2016, **351:**867-871.
